# A combined radiomics and anatomical features model enhances MRI-based recognition of symptomatic nerves in primary trigeminal neuralgia

**DOI:** 10.3389/fnins.2024.1500584

**Published:** 2024-10-24

**Authors:** Hongjian Li, Bing Li, Chuan Zhang, Ruhui Xiao, Libing He, Shaojie Li, Yu-Xin Yang, Shipei He, Baijintao Sun, Zhiqiang Qiu, Maojiang Yang, Yan Wei, Xiaoxue Xu, Hanfeng Yang

**Affiliations:** ^1^Department of Radiology, Affiliated Hospital of North Sichuan Medical College, Nanchong, China; ^2^North Sichuan Medical College Medical Imaging College, Nanchong, China; ^3^Department of Neurosurgery, The Second Affiliated Hospital of Fujian Medical University Quanzhou, Fujian, China; ^4^Beijing United Imaging Intelligent Imaging Technology Research Institute, Beijing, Beijing, China; ^5^Department of Pain Medicine, Affiliated Hospital of North Sichuan Medical College, Nanchong, Shanxi, China

**Keywords:** radiomics, multiple-ROI, MRI, trigeminal neuralgia, nomogram

## Abstract

**Background:**

The diagnosis of primary trigeminal neuralgia (PTN) in radiology lacks the gold standard and largely depends on the identification of neurovascular compression (NVC) using magnetic resonance imaging (MRI) water imaging sequences. However, relying on this imaging sign alone often fails to accurately distinguish the symptomatic side of the nerve from asymptomatic nerves, and may even lead to incorrect diagnoses. Therefore, it is essential to develop a more effective diagnostic tool to aid radiologists in the diagnosis of TN.

**Purpose:**

This study aims to establish a radiomics-based machine learning model integrating multi-region of interest (multiple-ROI) MRI and anatomical data, to improve the accuracy in differentiating symptomatic from asymptomatic nerves in PTN.

**Methods:**

A retrospective analysis of MRI data and clinical anatomical data was conducted on 140 patients with clinically confirmed PTN. Symptomatic nerves of TN patients were defined as the positive group, while asymptomatic nerves served as the negative group. The ipsilateral Meckel’s cavity (MC) was included in both groups. Through dimensionality reduction analysis, four radiomics features were selected from the MC and 24 radiomics features were selected from the trigeminal cisternal segment. Thirteen anatomical features relevant to TN were identified from the literature, and analyzed using univariate logistic regression and multivariate logistic regression. Four features were confirmed as independent risk factors for TN. Logistic regression (LR) models were constructed for radiomics model and clinical anatomy, and a combined model was developed by integrating the radiomics score (Rad-Score) with the clinical anatomy model. The models’ performance was evaluated using receiver operating characteristic curve (ROC) curves, calibration curves, and decision curve analysis (DCA).

**Results:**

The four independent clinical anatomical factors identified were: degree of neurovascular compression, site of neurovascular compression site, thickness of the trigeminal nerve root, and trigeminal pons angle (TPA). The final combined model, incorporating radiomics and clinical anatomy, achieved an area under the curve (AUC) of 0.91/0.90 (95% CI: 0.87–0.95/0.81–0.96) and an accuracy of approximately 82% in recognizing symptomatic and normal nerves.

**Conclusion:**

The combined radiomics and anatomical model provides superior recognition efficiency for the symptomatic nerves in PTN, offering valuable support for radiologists in diagnosing TN.

## 1 Introduction

Trigeminal neuralgia (TN) is a prevalent chronic neuropathic pain disorder with a lifetime prevalence ranging from 0.16 to 0.3%. It is more common in females and typically manifests between the ages of 53 and 57. TN is characterized by sudden, severe, electric shock-like or stabbing pain in areas innervated by the unilateral trigeminal nerve. These pain attacks may be triggered by minimal stimuli, or occur spontaneously (Cruccu et al., 2020; Bendtsen et al., 2020).

TN is classified into primary trigeminal neuralgia (PTN) and secondary trigeminal neuralgia (STN). STN is usually associated with identifiable neurological conditions, such as multiple sclerosis or space-occupying lesions, making its diagnosis is relatively straightforward. In contrast, the etiology of PTN is less clear, complicating its diagnosis. While NVC is a widely accepted theory for PTN, but not all PTN patients exhibit NVC, and NVC is also found in asymptomatic individuals, suggesting that it is not be the sole pathogenic factor of PTN (Cruccu et al., 2020; Adamczyk et al., 2007; Ashina et al., 2024).

Recent studies have identified localized microdemyelination in PTN patients have localized micro demyelination, which may be associated with structural and functional changes in the brain. These microdemyelinations are not always caused by NVC (Ashina et al., 2024; Wang et al., 2019). Additionally, variations in the Meckel’s cavity (MC) have been implicated in TN. For example, in percutaneous balloon compression (PBC) treatments for TN, pain relief depends on the shape and pressure exerted by the balloon on the trigeminal ganglion within the MC. Morphological differences in the MC between the Meckel cavity of TN patients and healthy individuals have also been reported (Lin et al., 2021; Jain et al., 2021; Wang et al., 2021; Li et al., 2021). Therefore, we have reason to suspect that there are differences in MC of TN patients compared with normal people. These differences may be multifaceted, such as differences in morphology, symmetry, and even nerve roots within MC. These differences may be independent of NVC.

The diagnosis of PTN is challenging, as it can be mistaken for other causes of facial pain, such as toothache or migraine. MRI of the trigeminal nerve is commonly used to aid in diagnosis, but radiologists often rely solely on identifying NVC. This method is not always accurate, as texture differences or microdemyelination in the trigeminal cistern segment, or morphological changes in the MC may be too subtle to detect with the naked eye, even with high-resolution MRI. For example, Magown et al. (2019) study found that 32% of TN patients may not have NVC, while Kress et al. (2006) also found that a large number of normal individuals have NVC in their trigeminal nerves. Therefore, there is crucial to find a pressing need for more effective diagnostic tools that can assist radiologists in identifying PTN, thus improving diagnostic accuracy and advancing our understanding of TN pathogenesis.

Radiomics, a non-invasive approach that extracts a large number of quantitative information features from medical images, has primarily been used in oncology but is now being explored in neuroimaging and pain disorders. Radiomics can capture subtle morphological changes, signal intensity variations, and texture differences that may go unnoticed by the naked eye. Previous studies have also highlighted anatomical differences in TN patients are also different from normal people, such as variations in TPA and trigeminal nerve root thickness, which could be diagnostically significant when incorporated into a machine learning model (Barzaghi et al., 2020).

This study focuses on PTN patients, and aims to: (1) Develop a radiomics model using the MC and trigeminal cisternal segment as the target areas, and a clinical anatomical model was established based on the anatomical features. These models are then combined to create an enhanced diagnostic tool. (2) Evaluate the accuracy of identifying symptomatic nerves using NVC signs alone, verifing that NVC is not a necessary and sufficient condition for PTN and simulating the scenario where radiologists rely on visual assessment of NVC during routine diagnosis.

## 2 Materials and methods

### 2.1 Patients

This study was carried out in accordance with the declaration of Helsinki and was approved by the Ethics Committee of the hospital. The study included 140 patients with TN clinically diagnosed with TN between January 2021 and April 2023. Among the 140 patients, 139 had unilateral trigeminal neuralgia and 1 had bilateral trigeminal neuralgia, so a total of 141 symptomatic nerves and 139 normal nerves were included. One of the normal nerves was excluded due to the lack of clarity, so a total of 279 nerves were included, including 141 symptomatic nerves and 138 normal nerves.

The study comprised 141 symptomatic nerves were included in the positive group (one patient had bilateral trigeminal onset) classified as the positive group, and 138 normal nerves classified as the negative group, totaling 279 nerves. Additionally, 141 MCs ipsilateral to the nerve were included in the positive group, and 138 MCs were included in the negative group, a total of 279 nerves (one patient’s normal nerve was excluded due to unclear display). A total of 141 MCs on the ipsilateral side of the nerve were included as the positive group, and 138 MCs were negative group. MCs were also included in the same way. A total of 279 MCs were included, including 141 on the symptomatic side and 138 on the normal side.

Inclusion criteria: (1) TN patients diagnosed according to the third edition of the International Classification of Headache Disorders. (2) The MRI sequence used was t2-mix3d-tra-spair. (3) Because the nerve root is very thin, the slice thickness of MRI must be as thin as possible (≤5 mm), covering the range includes MC and trigeminal cisternal segment. (4) Patients without prior trigeminal nerve-related surgery. Exclusion criteria: (1) TN patients with unclear localization of symptomatic nerves and normal nerves. (2) Patients with poor MRI image quality. (3) Patients diagnosed with STN or conditions that could cause secondary TN, such as herpes zoster infection, multiple sclerosis, or trigeminal schwannoma.

Our nerves were randomly divided into training and validation groups at a 7:3 ratio. One of the two nerves of a patient may be located in the training group, the other in the validation group, or the two nerves may be in the same group at the same time.

### 2.2 MRI water imaging parameters

Preoperative MRI scans were performed using a 3.0 T United Imaging MRI scanner (Discover uMR 790). The MRI sequence uses a specific neural water imaging sequence used was t2-mix3d-tra-spair, with the following parameters are as follows: TR = 1,300 ms; TE = 258 ms; FOV = 180 × 200 mm; scan slice thickness = 0.5 cm; voxel size = 0.5 × 0.5 × 0.5 cm.

### 2.3 Identification of NVC

NVC identification of nerve was conducted by two radiologists with 10 years of experience in diagnosing neurological/pain disorders who were blinded to the symptomatic side of the patients during analysis. If they have differences, the third professor with 27 years of experience in neuroimaging diagnosis will resolve them.

**Figure 1:**
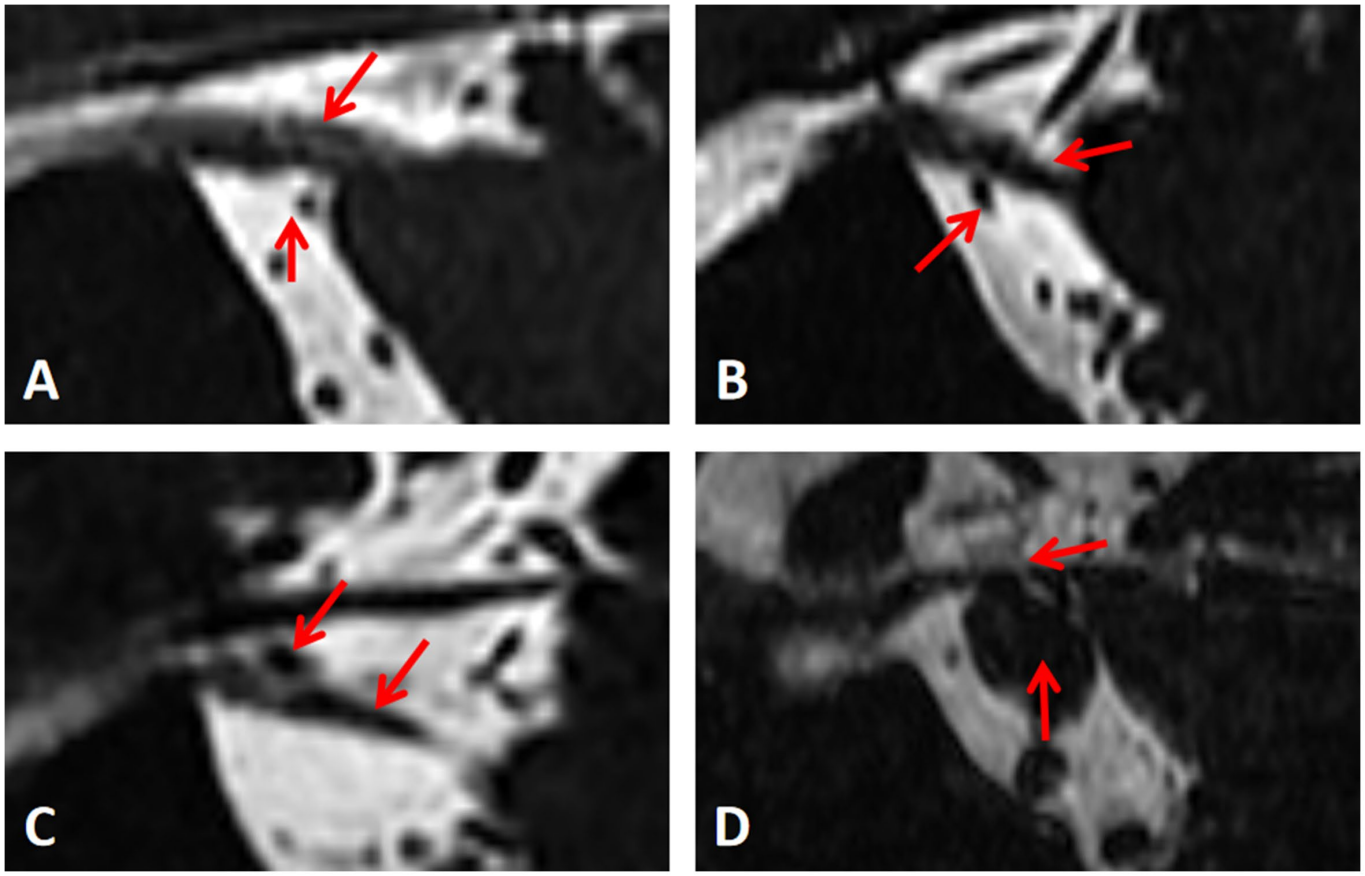
Measurement of the degree of neurovascular compression. **(A-D)** Represent level I, II, III and IV respectively.

We classify NVC was classified into four levels: Level I: Separation—blood vessels are distant from nerves, with cerebrospinal fluid (CSF) signal visible between them. Level II: Contact —nerve and blood vessel are in close proximity, without a CSF signal shadow between them. Level III: Compression—blood vessels compress the nerve, causing indentation and local tortuosity. Level IV: Shifting—severe compression by blood vessels results in nerve displacement, atrophy, and thinning. These levels are illustrated in Figure 1. For subsequent analysis, Level I was considered NVC-negative, while Levels II-IV were deemed NVC-positive, simulating radiologists’ reliance on visual assessment of NVC for diagnosing symptomatic nerves in actual work, that is, the nerves with NVC positive are judged as symptomatic nerves, and the nerves with NVC negative are normal nerves (Hung et al., 2017; Baroni et al., 2023).

### 2.4 Measurement of other clinical anatomic indicators

**Figure 2:**
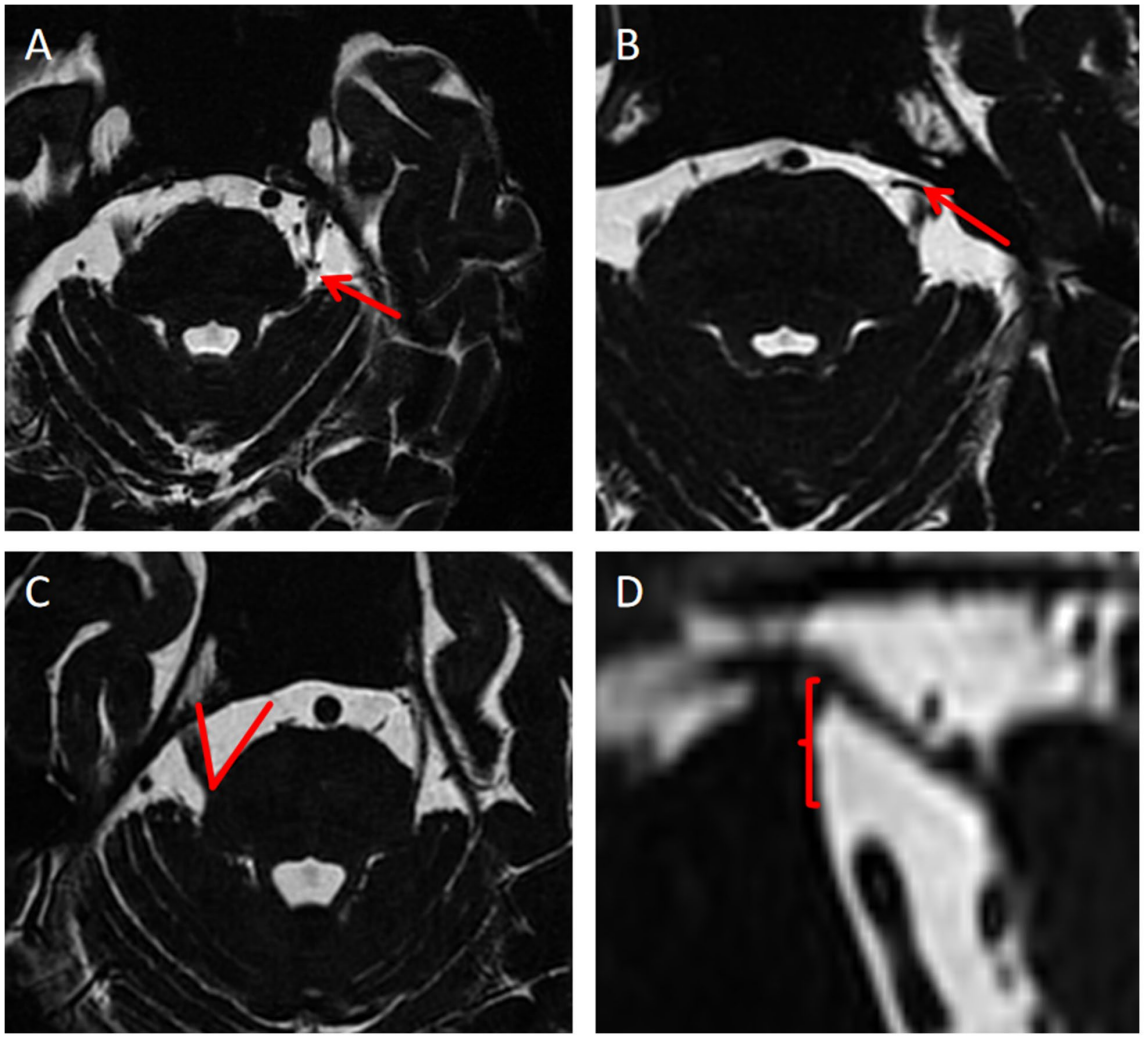
Measurement methods of some anatomical indexes. **(A)** Neurovascular compression or the adjacent site is distal; **(B)** Neurovascular compression or the adjacent site is proximal; **(C)** Measurement of TPA; **(D)** Nerve vertical height.

Thirteen anatomical indicators, identified from the literature as potentially differing between TN patients and healthy individuals, were selected. These indicators included the degree and site of NVC, neurovascular compression or adjacent sites (the compression point is proximal or distal) (Zhao et al., 2022), thickness of responsible blood vessels, maximum MC diameter of MC, maximum transverse MC diameter of MC, maximum MC cross-sectional area, trigeminal nerve root thickness (Li et al., 2024), length of trigeminal nerve cistern segment length, area of the anterior pool of the cerebral bridge, nerve vertical height (Li et al., 2024; Kundakci et al., 2022), TPA, angle of the petrous ridge (APR), and trigeminal nerve angles (ATN) (Hung et al., 2017; Barzaghi et al., 2021; Brinzeu et al., 2018; Zhong et al., 2022). Figure 2 shows the measurement methods for some anatomical indices. Univariate logistic regression analysis and multivariate logistic regression analyses were used to screen these anatomical characteristics, identifying independent risk factors for TN. were selected.

### 2.5 Image segmentation and radiomics feature extraction

**Figure 3:**
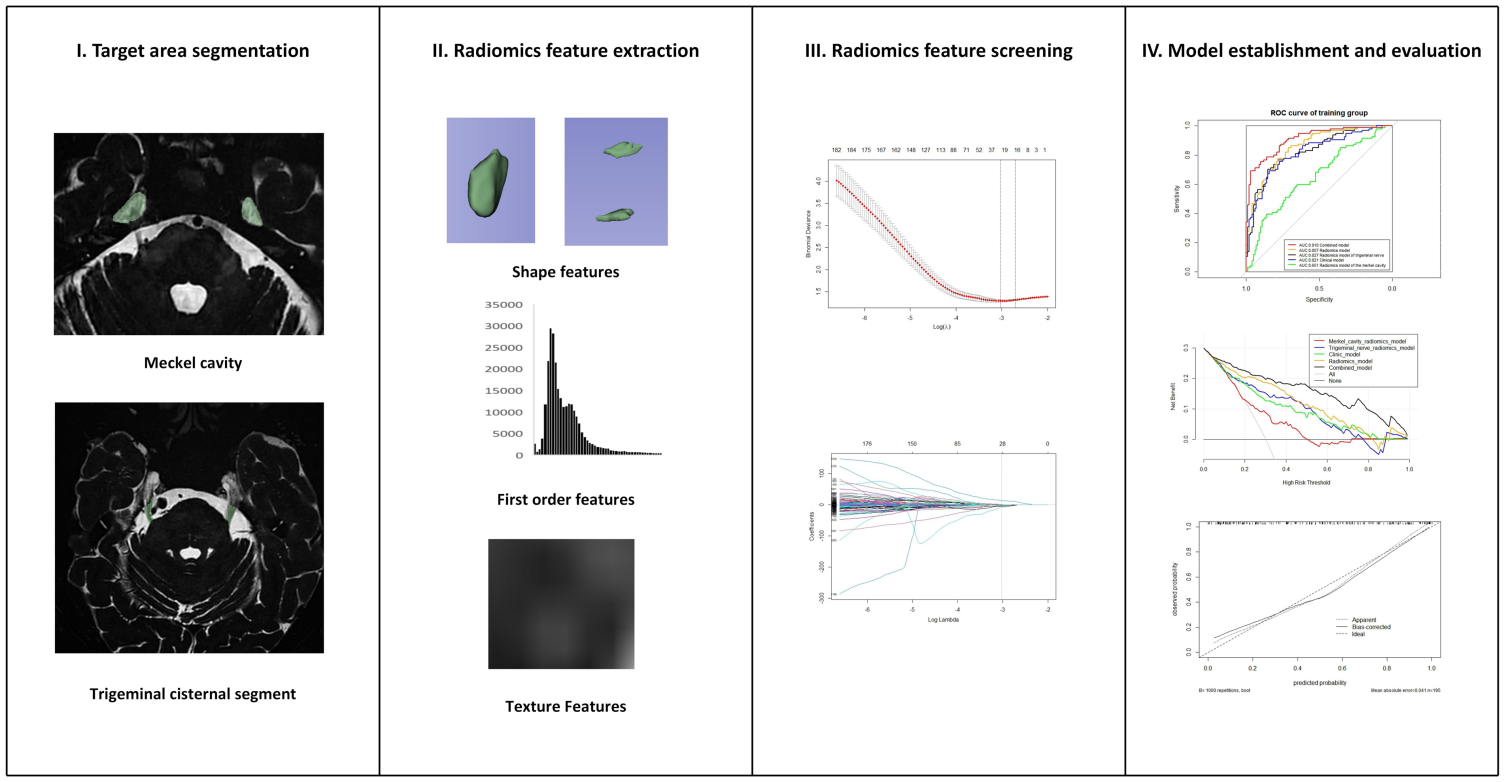
Research steps of radiomics.

The research steps of radiomics are shown in Figure 3. Image segmentation was performed using 3D Slicer (Version 5.2.1)^1^ and its radiomics plugin. Feature extraction and model establishment of models were conducted using R language software (Version 4.2.2).^2^

A radiologist with a 10 years of neuroimaging experience, blinded to patient clinical histories, conducted the image segmentation. All images were resampled all images to a voxel size of 1 × 1 × 1 mm^3^ using B-spline i 5 mm 5 mm nterpolation, and then normalized. Two regions of interest (ROIs) were defined: the trigeminal cisternal segment, which starts from the origin of the trigeminal nerve origin at the pons to the entrance of the MC; and the MC itself, including all its contents. The ROIs were delineated layer by layer on the cross-sectional images to reconstruct the volume of interest (VOI). For each VOI, 1,316 radiomics features were extracted, including morphological features, texture, and higher-order features. Intra-observer reproducibility and measure the stability of radiomics characteristics were evaluated by re-segmenting the VOI in 20 randomly selected patients after 1 month. The VOI of 20 randomly selected patients was re segmented by another professor in the field of pain imaging in our department of radiology to assess inter observer reproducibility and stability.

All features were standardized using Z-scores, and those with high stability were retained, ICC (Intra-and inter correlation coefficient) > 0.75. The Least Absolute Shrinkage and Selection Operator (LASSO) regression model with 10-fold cross-validation was used to select non-zero coefficient features. The Rad-Score for each case was calculated by using the formula Rad-Score = radiomics feature value × coefficient + intercept (Park et al., 2020; Zhang et al., 2024).

### 2.6 Model construction and evaluation

The prediction model was constructed using R language software. Radiomics features extracted from the two VOIS of MC and trigeminal cisternal segment were used to build separate radiomics models, which were then combined to create a new radiomics model, with Rad-Score calculated. Independent risk factors for TN were used to construct a clinical model, and the clinical model was combined with Rad-Score to create a final model. A nomogram was drawn based on the combined model. Due to its simplicity and wide application in radiomics, logistic regression (LR) was employed to construct all models in this study.

The performance of each model was evaluated using ROC curves, with Delong tests used to analyze the differences in performance. Clinical applicability was assessed using DCA curves, and model calibration was evaluated using a calibration curves.

### 2.7 Statistical analysis

All statistical analyses were conducted using SPSS (version 26.0, IBM Corp., Armonk, NY) and R. Normally distributed continuous variables were analyzed using two independent sample t-tests, while non-normally distributed variables were analyzed using Mann–Whitney U tests. Categorical data were analyzed using chi-square test or Fisher’s exact tests, with *p* < 0.05 considered statistically significant. The “glmnet” package in R was used for LASSO regression, “pROC” for plotting ROC curves and calculating AUC values, “rms” for plotting nomograms and calibration curves, and “dcurves” for DCA plotting DCA.

## 3 Results

### 3.1 Screening results of clinical anatomical features

Baseline patient data of patients are detailed in Table 1. Univariate and multivariate logistic regression analyses identified four independent risk factors for PTN: degree of neurovascular compression, site of neurovascular compression, thickness of trigeminal nerve root, and TPA.

**Table 1:** Clinical characteristics of patients.

	Overall	Normal nerves	Symptomatic nerves	*p*
n	279	138	141	
Age	63.78 (12.64)			
Sex (F/M)	161/118			
Training group	195	101	94	
validation group	84	37	47	
Maximum diameter of Mel cavity [mean (*SD*)]	13.20 (2.15)	13.44 (2.16)	12.96 (2.13)	0.063
Maximum transverse diameter of Mel cavity [mean (*SD*)]	5.20 (1.14)	5.30 (1.18)	5.10 (1.10)	0.147
Maximum cross-sectional area of the Mel cavity [mean (*SD*)]	0.59 (0.20)	0.61 (0.21)	0.57 (0.20)	0.095
Thickness of trigeminal nerve root [mean (SD)]	422.04 (321.20)	469.25 (343.93)	375.83 (291.14)	0.015
Length of trigeminal nerve cistern segment [mean (*SD*)]	11.26 (2.11)	11.39 (2.05)	11.13 (2.17)	0.298
TPA [mean (*SD*)]	48.94 (12.61)	51.29 (12.03)	46.65 (12.78)	0.002
APR [mean (*SD*)]	100.02 (15.13)	103.95 (14.93)	96.18 (14.36)	<0.001
ATN [mean (*SD*)]	145.50 (14.83)	148.04 (13.94)	143.01 (15.29)	0.004
Area of the anterior pool of the cerebral bridge [mean (*SD*)]	2.18 (0.67)	2.16 (0.65)	2.20 (0.70)	0.595
Nerve vertical height [mean (SD)]	2.83 (4.95)	2.60 (5.07)	3.07 (4.84)	0.43
Degree of vascular compression (%)				<0.001
1	45 (16.1)	34 (24.6)	11 (7.8)	
2	60 (21.5)	37 (26.8)	23 (16.3)	
3	105 (37.6)	50 (36.2)	55 (39.0)	
4	69 (24.7)	17 (12.3)	52 (36.9)	
Neurovascular compression site = 0/1 (%) (0 for near segment, 1 for far segment)	165/114 (59.1/40.9)	54/84 (39.1/60.9)	111/30 (78.7/21.3)	<0.001
Thickness of responsible blood vessels (%)				0.002
1	54 (19.4)	37 (26.8)	17 (12.1)	
2	167 (59.9)	80 (58.0)	87 (61.7)	
3	58 (0.8)	21 (15.2)	37 (26.2)	

### 3.2 Screening results of radiomics features

**Figure 4:**
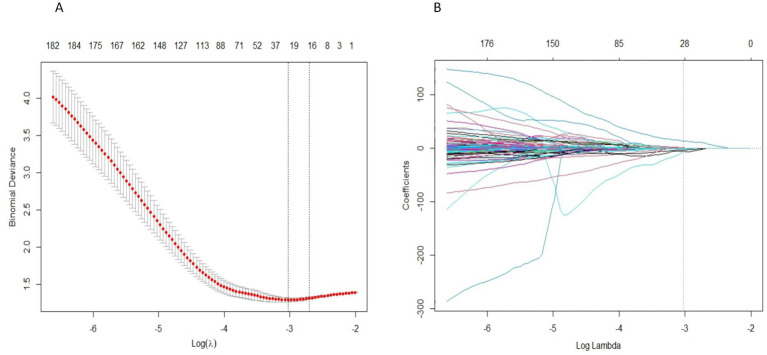
Radiomics feature selection uses LASSO regression. **(A)** The LASSO coefficient of radiomics features converges. **(B)** Penalty parameter (Lambda) selection, using the minimum deviation standard for 10 fold cross validation. The dashed line represents the optimal value of the penalty parameter (log (Lambda) = -3.0265) selected based on the minimum mean squared error criterion, resulting in 28 non-zero coefficient features.

A total of 2,632 radiomics features were extracted from the two ROIs, encompassing 28 shape features, 504 first-order statistical features, 672 GLCM features, 392 GLDM features, 448 GLRLM features, 448 GLSZM features, and 140 NGTDM features. After filtering for high stability (ICC > 0.75), 2,461 features were retained. LASSO regression further narrowed this down to screen, and a total of 28 radiomics features were selected, including 4 from the MC and 24 from the trigeminal cisternal segment. The specific process of LASSO regression process is illustrated in Figure 4. Table 2 provides details about the selected radiomics features.

**Table 2:** Basic information of radiomics characteristics.

No.	ROI	The name of the radiomics feature	Coefficient
N1	–	(Intercept)	5.34E-01
N2	Meckel cavity	Firstorder (Mean)	1.58E-03
N3	Meckel cavity	Firstorder (Median)	2.53E-04
N4	Meckel cavity	Firstorder (Kurtosis)	9.90E-02
N5	Meckel cavity	Firstorder (Skewness)	1.21E-01
N6	Cisternal segment of trigeminal nerve	Glszm (SmallAreaLowGrayLevelEmphasis)	−7.17E+00
N7	Cisternal segment of trigeminal nerve	Shape (Sphericity)	−1.27E+00
N8	Cisternal segment of trigeminal nerve	Firstorder (RootMeanSquared)	−1.12E-03
N9	Cisternal segment of trigeminal nerve	Glrlm (RunVariance)	−6.22E+00
N10	Cisternal segment of trigeminal nerve	Glszm (LargeAreaEmphasis)	−5.89E-02
N11	Cisternal segment of trigeminal nerve	Glcm (JointAverage)	−2.67E-03
N12	Cisternal segment of trigeminal nerve	Glcm (SumAverage)	−1.74E-15
N13	Cisternal segment of trigeminal nerve	Ngtdm (Coarseness)	1.34E+01
N14	Cisternal segment of trigeminal nerve	Firstorder (Kurtosis)	1.37E-02
N15	Cisternal segment of trigeminal nerve	Firstorder (Minimum)	−6.89E-05
N16	Cisternal segment of trigeminal nerve	Firstorder (Range)	9.12E-05
N17	Cisternal segment of trigeminal nerve	Glcm (Idn)	4.89E-01
N18	Cisternal segment of trigeminal nerve	Firstorder (Minimum)	−2.73E-04
N19	Cisternal segment of trigeminal nerve	Glszm (LargeAreaLowGrayLevelEmphasis)	−7.78E-01
N20	Cisternal segment of trigeminal nerve	Ngtdm (Busyness)	−3.43E+00
N21	Cisternal segment of trigeminal nerve	Glszm (SizeZoneNonUniformityNormalized)	4.21E-02
N22	Cisternal segment of trigeminal nerve	Ngtdm (Contrast)	4.90E-05
N23	Cisternal segment of trigeminal nerve	Firstorder (Energy)	2.51E-10
N24	Cisternal segment of trigeminal nerve	Firstorder (TotalEnergy)	3.02E-10
N25	Cisternal segment of trigeminal nerve	Gldm (LargeDependenceHighGrayLevelEmphasis)	6.92E-05
N26	Cisternal segment of trigeminal nerve	Firstorder (Variance)	3.28E-08
N27	Cisternal segment of trigeminal nerve	Gldm (GrayLevelVariance)	7.10E-05
N28	Cisternal segment of trigeminal nerve	Glrlm (GrayLevelVariance)	7.58E-06
N29	Cisternal segment of trigeminal nerve	Glszm (GrayLevelVariance)	6.08E-04

### 3.3 Simulating radiologists’ visual recognition of symptomatic nerves

**Figure 5:**
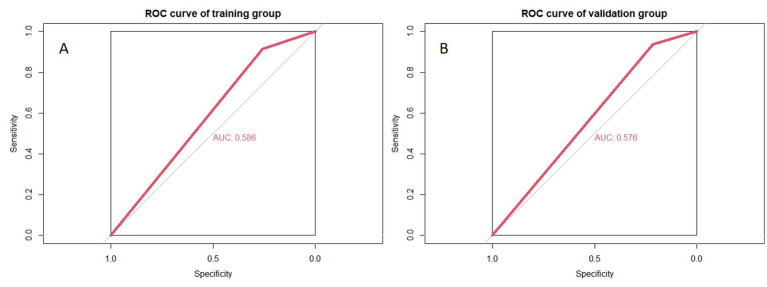
Accuracy of simulating radiologists’ visual recognition of symptomatic nerves. **(A,B)** Represent the training and validation groups, respectively.

To simulate the diagnostic process used by radiologists, a machine learning model was constructed using only NVC as a variable. In this model, NVC was treated as a binary variable, where any nerve with NVC was considered symptomatic nerve, and nerves without NVC were considered normal nerve. The ROC curve for this model is shown in Figure 5, demonstrating its limited diagnostic performance.

### 3.4 Performance of the final model

**Figure 6:**
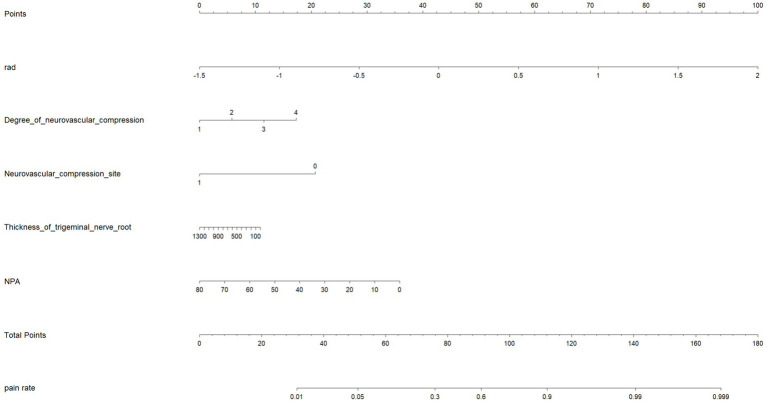
Nomogram for identifying PTN symptom nerves.

The four radiomics features from the MC were used to construct the Meckel’s cavity radiomics model, while the 24 radiomics features from the trigeminal cisternal segment were used to construct the trigeminal radiomics model. The performance of these models is summarized in Table 3. By combining the radiomics features from both ROIs, a more robust radiomics model was constructed. The four independent clinical anatomical features were used to build a clinical model, which was then combined with Rad-Score to form the final model. The nomogram based on the combined model is shown in Figure 6. Among all variables in the nomogram, Rad-Score had the greatest impact on diagnostic performance, emphasizing the importance of the radiomics model. The performance of the combined model. The results of these three models in both the training and validation groups is detailed in Table 4. All 5 models were built using LR.

**Table 3:** Performance of Meckel’s cavity radiomics model and trigeminal nerve radiomics model.

Training group/Validation group (95%CI)		
	Radiomics model of trigeminal nerve	Radiomics model of the Meckel’s cavity
AUC	0.83 (0.77–0.88)/0.78 (0.68–0.88)	0.65 (0.57–0.73)/0.69 (0.57–0.80)
ACC	0.77 (0.71–0.83)/0.73 (0.62–0.82)	0.63 (0.55–0.69)/0.63 (0.52–0.73)
SEN	0.76 (0.66–0.84)/0.68 (0.53–0.81)	0.51 (0.41–0.62)/0.60 (0.44–0.74)
SPE	0.79 (0.70–0.87)/0.78 (0.62–0.90)	0.73 (0.64–0.82)/0.68 (0.50–0.82)
PPV	0.77 (0.69–0.83)/0.80 (0.68–0.88)	0.64 (0.55–0.72)/0.70 (0.58–0.80)
NPV	0.78 (0.71–0.83)/0.66 (0.55–0.75)	0.62 (0.56–0.67)/0.57 (0.47–0.67)
YI	0.55/0.46	0.24/0.28

AUC, area under the curve; ACC, accuracy; SEN, sensitivity; SPE, specificity; PPV, positive predictive value; NPV, negative predictive value; YI, Youden’s index.

**Table 4:** Performance of clinical model, radiomics model, and combined model.

Training group/Validation group (95%CI)			
	Clinical model	Radiomics model	Combined model
AUC	0.81 (0.76–0.88)/0.78 (0.66–0.87)	0.86 (0.81–0.91)/0.87 (0.79–0.95)	0.91 (0.87–0.95)/0.90 (0.81–0.96)
ACC	0.72 (0.65–0.78)/0.68 (0.56–0.78)	0.77 (0.70–0.83)/0.80 (0.70–0.88)	0.82 (0.75–0.87)/0.82 (0.72–0.90)
SEN	0.71 (0.61–0.80)/0.67 (0.50–0.80)	0.74 (0.64–0.83)/0.72 (0.57–0.84)	0.82 (0.73–0.89)/0.82 (0.69–0.92)
SPE	0.72 (0.62–0.80)/0.69 (0.51–0.83)	0.79 (0.70–0.87)/0.89 (0.75–0.97)	0.81 (0.72–0.88)/0.81 (0.65–0.92)
PPV	0.71 (0.63–0.77)/0.70 (0.58–0.80)	0.77 (0.69–0.83)/0.89 (0.77–0.96)	0.80 (0.73–0.86)/0.85 (0.74–0.92)
NPV	0.73 (0.66–0.79)/0.65 (0.53–0.75)	0.77 (0.70–0.83)/0.72 (0.61–0.80)	0.83 (0.76–0.88)/0.79 (0.66–0.88)
YI	0.43/0.36	0.53/0.61	0.63/0.63

**Figure 7:**
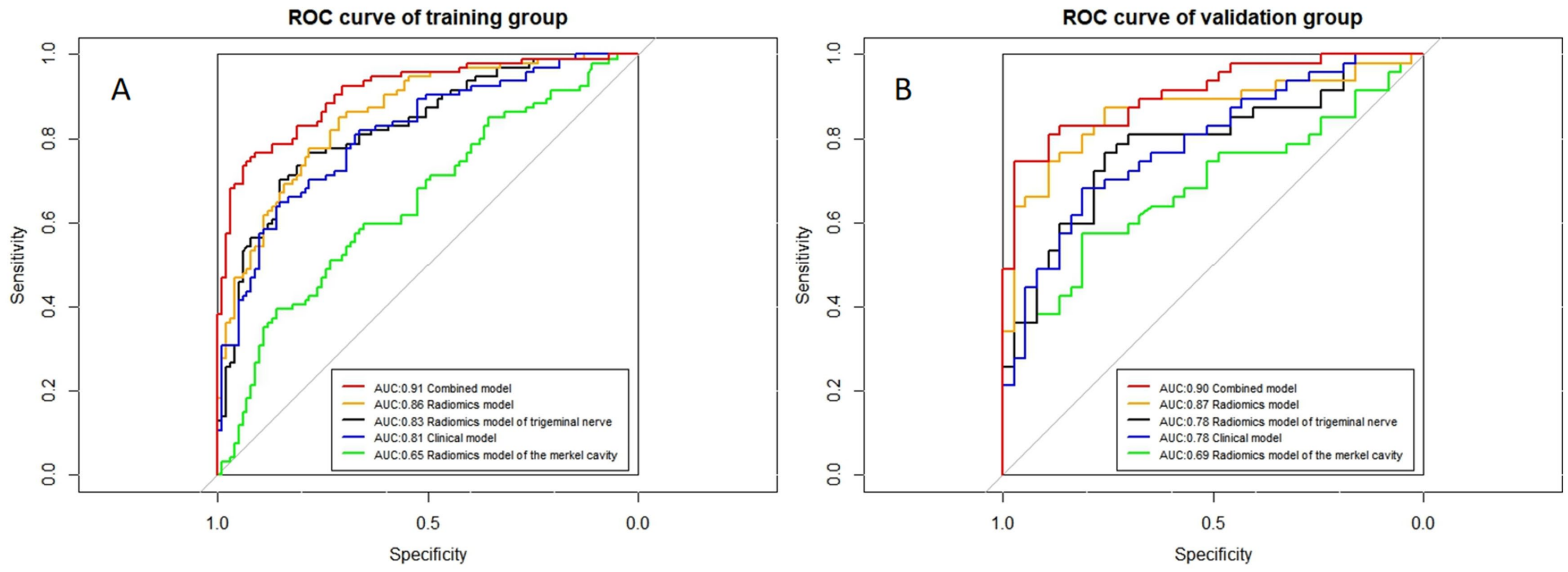
ROC curves of all models. **(A,B)** Represent the training and validation groups, respectively.

**Figure 8:**
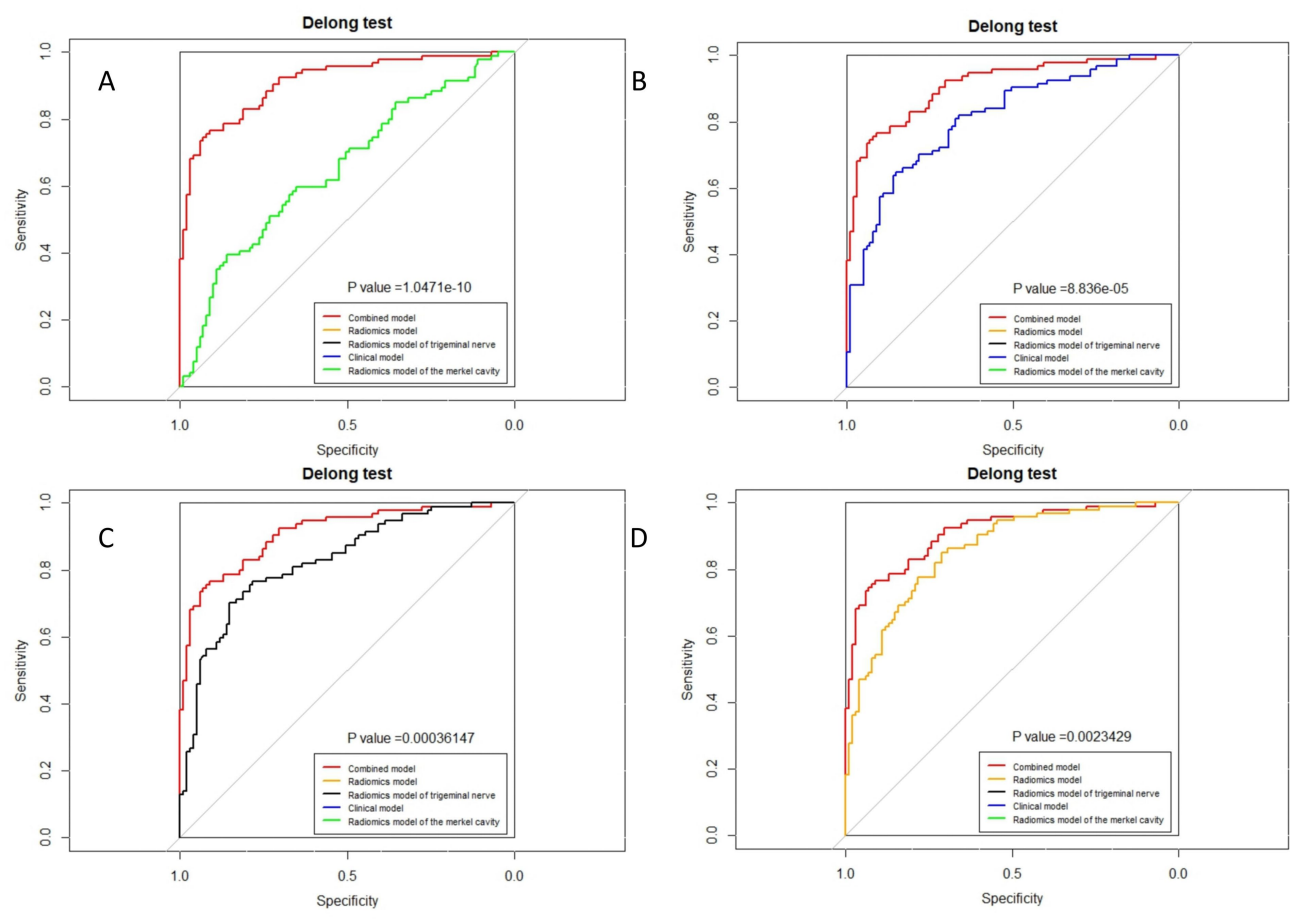
Delong test of combined model and other models in the training group. **(A)** Radiomics model of Meckel cavity; **(B)** Clinical model; **(C)** Radiomics model of trigeminal nerve; **(D)** Radiomics model.

**Figure 9:**
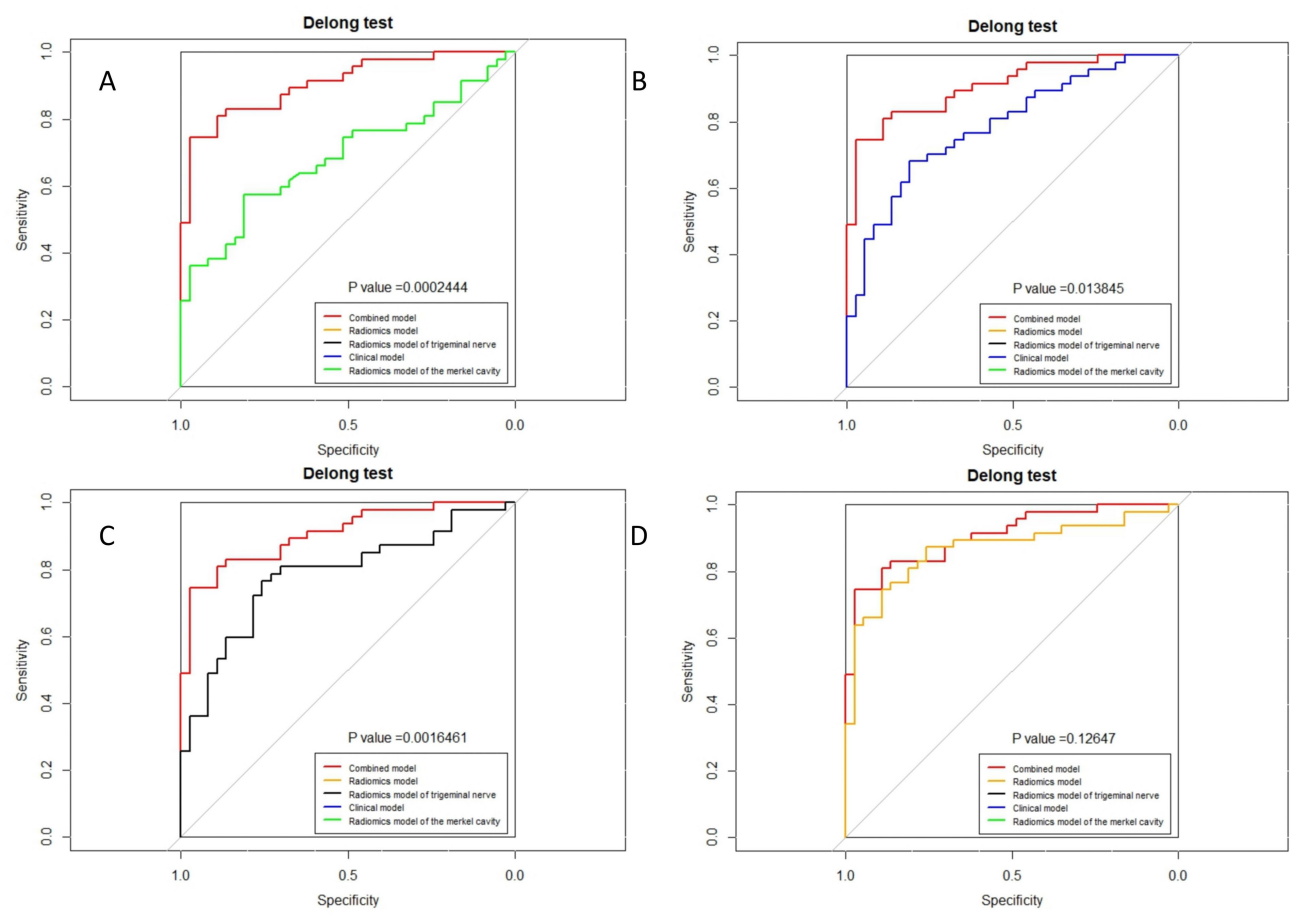
Delong test of combined model and other models in the validation group. **(A)** Radiomics model of Meckel cavity; **(B)** Clinical model; **(C)** Radiomics model of trigeminal nerve; **(D)** Radiomics model.

**Figure 10:**
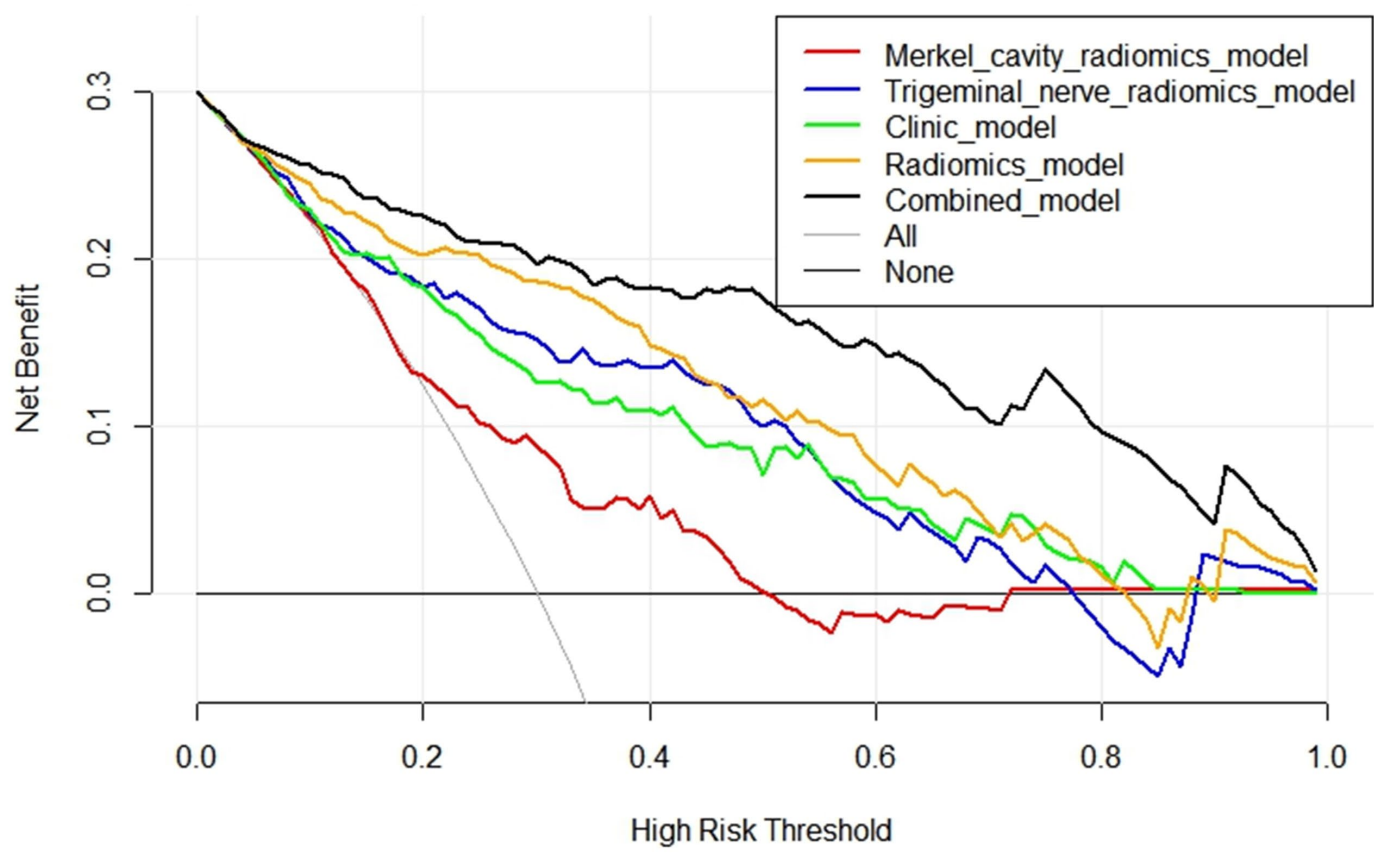
Comparison of DCA curves of five models.

**Figure 11:**
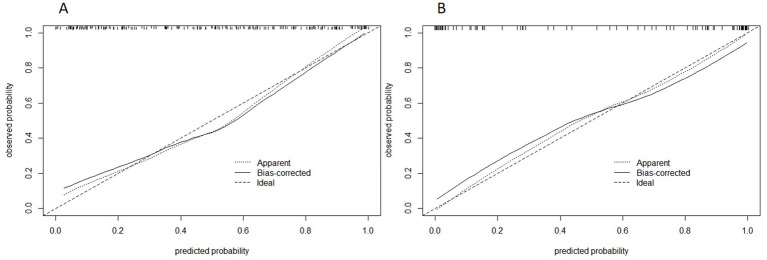
Calibration curve of combined model of training group **(A)** and validation group **(B)**. The abscissa is the predicted probability and the ordinate is the actual probability.

The ROC curves for all models are presented in Figure 7. Overall, the performance of the combined model outperformed the others, with the radiomics model being the second best, followed by the trigeminal radiomics model, the clinical model is the fourth, and finally the Meckel’s cavity radiomics model. The AUC values for the combined model reached 0.91 and 0.90 in the training and validation groups, respectively, which was significantly outperforming the performance of other models. With *p* < 0.05 as the standard, the Delong test was used to analyze whether the performance differences between the combined model and the radiomics model, the clinical model, the trigeminal radiomics model, and the Meckel cavityradiomics model were statistically significant. The Delong test results for both groups are shown in Figures 8, 9 indicating that the combined model significantly outperforms others (*p* < 0.05). The DCA curves (Figure 10) further support the superior clinical utility of the combined model. The calibration curves for the combined model in both groups (Figure 11) show. Of course, the accuracy of the combined model is also much greater than the accuracy of radiologists’ visual recognition of symptomatic nerves. The radiomics model also has stronger diagnostic performance than the MC radiomics model and trigeminal radiomics model alone (*p-*values were 0.0001/0.002, 0.07/0.01, respectively).

The DCA curves of the five models are shown in Figure 10. The black curve representing the combined model in the figure does not intersect with the horizontal axis and the vertical axis, indicating that the model has good clinical application ability. And in all abscissa ranges, the black curve is higher than any other curve, which means that the clinical value of the combined model is the highest among all models. Figure 11 shows the correction curves of the combined model in the training group and the validation group respectively, and both ends of the curve fit well with the ideal curve. The predicted probability of the combined model is in good agreement between predicted and actual probabilities.

## 4 Discussion

This study successfully developed a combined model integrating radiomics features extracted from the two ROIs of trigeminal nerve and Meckel’s cavity (MC) with clinical anatomical data to identify symptomatic nerves in primary trigeminal neuralgia (PTN). The model demonstrated robust diagnostic performance, significantly enhancing accuracy compared to traditional visual assessment methods.

### 4.1 Pathophysiological insights into PTN

The simulation of radiologists’ visual identification of symptomatic nerves based solely on neurovascular compression (NVC) showed poor accuracy, highlighting the limitations of this approach. Similar findings were reported by Darrow et al. (2022), where even senior neuroradiologists struggled to accurately identify symptomatic nerves by naked eyes is indeed limited, and radiologists have a high error rate. These results suggest that NVC alone is insufficient as a diagnostic criterion for PTN. Additional factors, such as minor anatomical changes or demyelination, likely play critical roles. This supports the hypothesis that NVC acts more as a trigger rather than a direct cause of PTN, with other factors such as minor anatomical alterations [e.g., trigeminal pons angle (TPA)] trigeminal neuritis, and microstructural changes in the trigeminal nerve contributing to the disease’s pathogenesis (Hutchins et al., 1990; Cheng et al., 2015). Many pathological studies have shown that the nerve roots with lesions exhibit tiny local demyelination changes (Love and Coakham, 2001). We speculate that factors such as vascular compression, trigeminal neuritis, minor anatomical changes (like those identified in this study), and lesions in the anterior pontine area could increase the excitability of demyelinated trigeminal nerve axons. These factors could further lead to the generation of spontaneous ectopic impulses and ephaptic transmission between nociceptive and non-nociceptive fibers, ultimately triggering pain (Mousavi et al., 2024; Mannerak et al., 2021). In addition to the morphological changes in the Meckel’s Cavity mentioned earlier, factors such as the absence of MC, encephalitis, abnormal enhancement of neural signals in MC due to viral infection, and persistent compression of ganglia by the trigeminal artery within MC may also act as triggering factors for trigeminal neuralgia (Jain et al., 2021; Wang et al., 2021; Li et al., 2021; Malhotra et al., 2018; Li et al., 2023).

This study identified four anatomical factors that can be used to establish a model. The degree of neurovascular compression is easy to understand, and the greater the degree of compression, the more likely it is to cause PTN. The compression of nerve vessels at the proximal end is more likely to cause PTN. Our hypothesis is that the area near the brainstem of the trigeminal nerve is the transition point from the central nervous system to the periphery, where NVC is more likely to cause demyelination changes (Garcia et al., 2012). The smaller the TPA, the easier it is for nerves to become symptomatic nerves. Our hypothesis is that the smaller the TPA, the easier it is for the trigeminal nerve root to come into contact with the arachnoid membrane around the brainstem and undergo demyelination changes. Finally, demyelination of the trigeminal nerve caused by various reasons can lead to atrophy and thinning of the nerve itself, resulting in PTN.

Therefore, we believe that the aforementioned triggering factors for PTN likely exist, but some factors may be too subtle to be easily recognized by the naked eye. It is theoretically feasible to diagnose PTN through radiomics, accurate measurement, and machine learning modeling. For instance, Lin et al. (2021) demonstrated the abnormality of flatness in the MC of PTN patients using radiomics. Our study also underscores the feasibility of using radiomics for PTN diagnosis, indirectly suggesting that NVC is not the cause of PTN, but rather a triggering factor. This opens new avenues for exploring the pathogenesis of PTN.

### 4.2 Developing imaging quantitative standards for PTN diagnosis

In this study, we selected some characteristics of PTN patients that may differ from normal individuals, and after screening, we ultimately identified four independent risk factors for PTN, such as TPA, which differ from those in normal individuals. Beyond radiomics, it is also possible to develop a set of diagnostic criteria based on clinical anatomical features that radiologists can use. These criteria, primarily based on anatomical parameters, can be easily measured using high-resolution MRI. Additionally, the models constructed based on these clinical anatomical features demonstrated high accuracy (AUC = 0.81/0.78) (Xu et al., 2024). Other features that can also be included in this set of imaging standards. Patients with PTN may exhibit changes in brain lobe morphology, solar pontine lesions (SPL), increased pontine volume, and variations in adjacent vascular structures (Kundakci et al., 2022; Zhang et al., 2022; Tang et al., 2020; Tohyama et al., 2020). In addition to water imaging, PTN patients also show changes in enhanced sequences, diffusion-weighted imaging sequences (DWI), diffusion tensor imaging (DTI), functional MRI (fMRI), and magnetic resonance spectroscopy sequences (MRS) sequences (Alper et al., 2017; Arda et al., 2021; Leal et al., 2019; Wu et al., 2020). Although some anatomical features did not show predictive value in this study, but it does not mean that they might still be related to PTN. Measurement errors and interrelationships between variables could explain their lack of significance. Therefore, features such as the angle of the petrous ridge (APR) and trigeminal nerve angles (ATN), which were not included in this study, could also be considered for future diagnostic criteria.

In general, there are many anatomical features that can help identify PTN symptomatic nerves in PTN. The key to developing a quantitative diagnostic standard for PTN imaging lies in selecting a few common features with strong diagnostic performance and simple measurement methods. Constructing a structured and quantitative imaging diagnostic template using these features could significantly improve radiologists’ diagnostic accuracy in PTN.

### 4.3 Speculation on the location of PTN etiology

This study selected two regions of interest (ROIs), the trigeminal cisternal segment and MC, were selected for radiomics analysis. The combined radiomics model outperformed models constructed from individual ROIs, suggesting that both the MC and trigeminal cisternal segment are potential sites of PTN pathogenesis. The higher performance of the trigeminal radiomics model suggests that the etiology may primarily reside in the trigeminal cisternal segment, which is also consistent with the current research (Gambeta et al., 2020). Although some studies have reported secondary changes in the peripheral branches of the trigeminal nerve in PTN patients, which are not easily observed by MRI (Marinkovic et al., 2009), the high accuracy of the radiomics model constructed in this study is high enough (AUC: 0.87) supports the focus on the MC and trigeminal cisternal segment in clinical practice.

### 4.4 Limitations and future directions

This study has several limitations. First of all, as a retrospective study, the sample size is relatively small, and it does not include a normal population. Efforts are underway to collect more patient data and include healthy volunteers for follow-up studies. Second, PTN patients with some etiologies located in peripheral branches (e.g., supraorbital neuralgia) may have been included in this study, but the selected ROIs did not encompass these peripheral branches. Excluding these patients may improve the model’s performance. Lastly, incorporating additional imaging sequences beyond water imaging (e.g., DTI, fMRI) and expanding the ROIs to include structures such as the midbrain aqueduct and striatum could yield meaningful results (Ge et al., 2023). Furthermore, incorporating additional anatomical features (e.g., characteristics of responsible vessels) can also be incorporated into clinical anatomical models could enhance their diagnostic utility (Holste et al., 2020).

## 5 Conclusion

Significant differences exist in the symptomatic side nerves, symptomatic side MCs, and some clinical anatomical features among PTN patients. These differences can be captured through radiomics or precise measurements. The machine learning model developed based on the differences in these indicators can accurately identifies symptomatic nerves in PTN patients, outperforming traditional visual diagnostic methods. This study provides an effective auxiliary tool for radiologists in diagnosing PTN and offers new insights into the pathological and physiological mechanisms of PTN.

## Data Availability

The raw data supporting the conclusions of this article will be made available by the authors, without undue reservation.

## Footnotes

^1^ http://www.slicer.org

^2^ http://www.r-project.org

## References

[ref1] Adamczyk M. Bulski T. Sowinska J. Furmanek A. Bekiesińska-Figatowska M. (2007). Trigeminal nerve - artery contact in people without trigeminal neuralgia - MR study. Med. Sci. Monit. 13, 38–43. 17507883

[ref2] Alper J. Shrivastava R. K. Balchandani P. (2017). Is there a magnetic resonance imaging-discernible cause for trigeminal neuralgia? A structured review. World Neurosurg. 98, 89–97. doi: 10.1016/j.wneu.2016.10.104, PMID: 27989975 PMC5326610

[ref3] Arda K. N. Akay S. Karadas O. Kartal O. (2021). Proton MR spectroscopic features of the cisternal segment of the trigeminal nerve in patients with trigeminal neuralgia: a pilot study. Clin. Imaging 74, 93–99. doi: 10.1016/j.clinimag.2020.12.006, PMID: 33465667

[ref4] Ashina S. Robertson C. E. Srikiatkhachorn A. di Stefano G. Donnet A. Hodaie M.. (2024). Trigeminal neuralgia. Nat. Rev. Dis. Primers 10:39. doi: 10.1038/s41572-024-00523-z 38816415

[ref5] Baroni S. Rapisarda A. Gentili V. Burattini B. Moretti G. Sarlo F.. (2023). CSF neuron-specific enolase as a biomarker of neurovascular conflict severity in drug-resistant trigeminal neuralgia: a prospective study in patients submitted to microvascular decompression. Neurol. Sci. 44, 1319–1325. doi: 10.1007/s10072-022-06573-z 36564658

[ref6] Barzaghi L. R. Albano L. Scudieri C. Gigliotti C. R. Nadin F. del Vecchio A.. (2020). Gamma knife radiosurgery for trigeminal neuralgia: role of trigeminal length and Pontotrigeminal angle on target definition and on clinical effects. World Neurosurg. 142, e140–e150. doi: 10.1016/j.wneu.2020.06.147, PMID: 32599193

[ref7] Barzaghi L. R. Albano L. Scudieri C. Gigliotti C. R. Nadin F. del Vecchio A.. (2021). Erratum to 'Gamma knife radiosurgery for trigeminal neuralgia: role of trigeminal length and Pontotrigeminal angle on target definition and on clinical Effects' [world neurosurgery. 142 (2020), e140-e150]. World Neurosurg. 149:512. doi: 10.1016/j.wneu.2021.02.035 33692007

[ref8] Bendtsen L. Zakrzewska J. M. Heinskou T. B. Hodaie M. Leal P. R. L. Nurmikko T.. (2020). Advances in diagnosis, classification, pathophysiology, and management of trigeminal neuralgia. Lancet Neurol. 19, 784–796. doi: 10.1016/S1474-4422(20)30233-7, PMID: 32822636

[ref9] Brinzeu A. Dumot C. Sindou M. (2018). Role of the petrous ridge and angulation of the trigeminal nerve in the pathogenesis of trigeminal neuralgia, with implications for microvascular decompression. Acta Neurochir. 160, 971–976. doi: 10.1007/s00701-018-3468-1, PMID: 29353407

[ref10] Cheng J. Lei D. Zhang H. Mao K. (2015). Trigeminal root compression for trigeminal neuralgia in patients with no vascular compression. Acta Neurochir. 157, 323–327. doi: 10.1007/s00701-014-2300-9 25572631

[ref11] Cruccu G. Di Stefano G. Truini A. (2020). Trigeminal Neuralgia. N. Engl. J. Med. 383, 754–762. doi: 10.1056/NEJMra1914484 32813951

[ref12] Darrow D. P. Mulford K. L. Quinn C. Spano A. Nixdorf D. R. Grande A.. (2022). The practical limits of high-quality magnetic resonance imaging for the diagnosis and classification of trigeminal neuralgia. Clin. Neurol. Neurosurg. 221:107403. doi: 10.1016/j.clineuro.2022.107403 35933966

[ref13] Gambeta E. Chichorro J. G. Zamponi G. W. (2020). Trigeminal neuralgia: an overview from pathophysiology to pharmacological treatments. Mol. Pain 16:174480692090189. doi: 10.1177/1744806920901890 PMC6985973 31908187

[ref14] Garcia M. Naraghi R. Zumbrunn T. Rösch J. Hastreiter P. Dörfler A. (2012). High-resolution 3D-constructive interference in steady-state MR imaging and 3D time-of-flight MR angiography in neurovascular compression: a comparison between 3T and 1.5T. AJNR Am. J. Neuroradiol. 33, 1251–1256. doi: 10.3174/ajnr.A2974, PMID: 22403774 PMC7965522

[ref15] Ge X. Wang L. Pan L. Ye H. Zhu X. Fan S.. (2023). Alteration of the cortical morphology in classical trigeminal neuralgia: voxel-, deformation-, and surface-based analysis. J. Headache Pain 24:17. doi: 10.1186/s10194-023-01544-x, PMID: 36809919 PMC9942396

[ref16] Holste K. Chan A. Y. Rolston J. D. Englot D. J. (2020). Pain outcomes following microvascular decompression for drug-resistant trigeminal neuralgia: a systematic review and meta-analysis. Neurosurgery 86, 182–190. doi: 10.1093/neuros/nyz075, PMID: 30892607 PMC8253302

[ref17] Hung P. S. Chen D. Q. Davis K. D.. (2017). Predicting pain relief: use of pre-surgical trigeminal nerve diffusion metrics in trigeminal neuralgia. Neuroimage Clin 15, 710–718. doi: 10.1016/j.nicl.2017.06.017, PMID: 28702348 PMC5491459

[ref18] Hutchins L. G. Harnsberger H. R. Jacobs J. M. Apfelbaum R. I. (1990). Trigeminal neuralgia (tic douloureux): MR imaging assessment. Radiology 175, 837–841. doi: 10.1148/radiology.175.3.2343134, PMID: 2343134

[ref19] Jain A. Muneer M. S. Okromelidze L. McGeary R. Valluri S. K. Bhatt A. A.. (2021). Absence of Meckel cave: a rare cause of trigeminal neuralgia. AJNR Am. J. Neuroradiol. 42, 1610–1614. doi: 10.3174/ajnr.A7205, PMID: 34244131 PMC8423049

[ref20] Kress B. Schindler M. Rasche D. Hähnel S. Tronnier V. Sartor K. (2006). Trigeminal neuralgia: how often are trigeminal nerve-vessel contacts found by MRI in normal volunteers. Rofo 178, 313–315. doi: 10.1055/s-2005-858959, PMID: 16508839

[ref21] Kundakci Y. E. Dogan N. U. Kara I.. (2022). Morphometric examination of trigeminal nerve and its adjacent structures in patients with trigeminal neuralgia: a case-control study. Turk. J. Med. Sci. 52, 1627–1638. doi: 10.55730/1300-0144.5503, PMID: 36422504 PMC10395698

[ref22] Leal P. Roch J. Hermier M. Berthezene Y. Sindou M. (2019). Diffusion tensor imaging abnormalities of the trigeminal nerve root in patients with classical trigeminal neuralgia: a pre-and postoperative comparative study 4 years after microvascular decompression. Acta Neurochir. 161, 1415–1425. doi: 10.1007/s00701-019-03913-5 31049710

[ref23] Li Y. Cao B. Wang Y. Shi H. du Y. Shi H.. (2024). Evaluation of the correlation between trigeminal nerve atrophy and trigeminal neuralgia using multimodal image fusion: a single-center retrospective study. Clin. Neurol. Neurosurg. 243:108387. doi: 10.1016/j.clineuro.2024.108387 38924844

[ref24] Li M. W. Jiang X. F. Niu C. S. (2021). Efficacy of and risk factors for percutaneous balloon compression for trigeminal neuralgia in elderly patients. Br. J. Neurosurg. 35, 280–284. doi: 10.1080/02688697.2020.1787341 32619112

[ref25] Li S. Liao C. Wu Y. Yang X. Zhang W. (2023). Association between morphological characteristics of Meckel's cave and outcomes after percutaneous balloon compression for primary trigeminal neuralgia. Neurosurg. Rev. 46:307. doi: 10.1007/s10143-023-02221-y, PMID: 37985480

[ref26] Li H. Zhang C. Yan W. Li Z. Liu Y. Sun B.. (2024). Radiomics nomogram based on MRI water imaging identifying symptomatic nerves of patients with primary trigeminal neuralgia: a preliminary study. Medicine (Baltimore) 103:e37379. doi: 10.1097/MD.0000000000037379, PMID: 38428849 PMC10906654

[ref27] Lin J. Zhang Y. Li W. Yan J. Ke Y. (2021). Flatness of the Meckel cave may cause primary trigeminal neuralgia: a radiomics-based study. J. Headache Pain 22:104. doi: 10.1186/s10194-021-01317-4 34479476 PMC8414677

[ref28] Love S. Coakham H. B. (2001). Trigeminal neuralgia: pathology and pathogenesis. Brain 124, 2347–2360. doi: 10.1093/brain/124.12.2347 11701590

[ref29] Magown P. Ko A. L. Burchiel K. J. (2019). The Spectrum of trigeminal neuralgia without neurovascular compression. Neurosurgery 85, E553–E559. doi: 10.1093/neuros/nyz048, PMID: 31329945

[ref30] Malhotra A. Tu L. Kalra V. B. Wu X. Mian A. Mangla R.. (2018). Neuroimaging of Meckel's cave in normal and disease conditions. Insights Imaging 9, 499–510. doi: 10.1007/s13244-018-0604-7, PMID: 29671218 PMC6108963

[ref31] Mannerak M. A. Lashkarivand A. Eide P. K. (2021). Trigeminal neuralgia and genetics: a systematic review. Mol. Pain 17:794217419. doi: 10.1177/17448069211016139 PMC8135221 34000891

[ref32] Marinkovic S. Gibo H. Todorovic V. Antić B. Kovacević D. Milisavljević M.. (2009). Ultrastructure and immunohistochemistry of the trigeminal peripheral myelinated axons in patients with neuralgia. Clin. Neurol. Neurosurg. 111, 795–800. doi: 10.1016/j.clineuro.2009.07.020, PMID: 19836877

[ref33] Mousavi S. H. Lindsey J. W. Westlund K. N. Alles S. R. A. (2024). Trigeminal neuralgia as a primary demyelinating disease: potential multimodal evidence and remaining controversies. J. Pain 25, 302–311. doi: 10.1016/j.jpain.2023.08.012, PMID: 37643657

[ref34] Park J. E. Kim H. S. Jo Y. Yoo R. E. Choi S. H. Nam S. J.. (2020). Radiomics prognostication model in glioblastoma using diffusion-and perfusion-weighted MRI. Sci. Rep. 10:4250. doi: 10.1038/s41598-020-61178-w, PMID: 32144360 PMC7060336

[ref35] Tang Y. Wang M. Zheng T. Yuan F. Yang H. Han F.. (2020). Grey matter volume alterations in trigeminal neuralgia: a systematic review and meta-analysis of voxel-based morphometry studies. Prog. Neuro-Psychopharmacol. Biol. Psychiatry 98:109821. doi: 10.1016/j.pnpbp.2019.109821, PMID: 31756417

[ref36] Tohyama S. Hung P. S. Cheng J. C. Zhang J. Y. Halawani A. Mikulis D. J.. (2020). Trigeminal neuralgia associated with a solitary pontine lesion: clinical and neuroimaging definition of a new syndrome. Pain 161, 916–925. doi: 10.1097/j.pain.0000000000001777, PMID: 31842151 PMC7170433

[ref37] Wang Q. Chen C. Guo G. Li Z. Huang D. Zhou H. (2021). A prospective study to examine the Association of the Foramen Ovale Size with intraluminal pressure of pear-shaped balloon in percutaneous balloon compression for trigeminal neuralgia. Pain Ther. 10, 1439–1450. doi: 10.1007/s40122-021-00311-7, PMID: 34460076 PMC8586299

[ref38] Wang Y. Yang Q. Cao D. Seminowicz D. Remeniuk B. Gao L.. (2019). Correlation between nerve atrophy, brain grey matter volume and pain severity in patients with primary trigeminal neuralgia. Cephalalgia 39, 515–525. doi: 10.1177/0333102418793643 30086682 PMC8889450

[ref39] Wu M. Qiu J. Jiang X. Li M. Wang S. D. Dong Q.. (2020). Diffusion tensor imaging reveals microstructural alteration of the trigeminal nerve root in classical trigeminal neuralgia without neurovascular compression and correlation with outcome after internal neurolysis. Magn. Reson. Imaging 71, 37–44. doi: 10.1016/j.mri.2020.05.006, PMID: 32439427

[ref40] Xu H. Liu Y. Zeng W. T. Fan Y. X. Wang Y. (2024). Distinctive cortical morphological patterns in primary trigeminal neuralgia: a cross-sectional clinical study. Neuroradiology 66, 207–216. doi: 10.1007/s00234-023-03257-z, PMID: 38001310

[ref41] Zhang F. Ni Y. Luo G. Zhang Y. Lin J. (2024). Independent association of the Meckel's cave with trigeminal neuralgia and development of a screening tool. Eur. J. Radiol. 171:111272. doi: 10.1016/j.ejrad.2023.111272, PMID: 38154423

[ref42] Zhang F. Zhang G. Luo H. Zhang Y. Lin J. (2022). Significance of different offending vessels and development of a potential screening tool for trigeminal neuralgia. Eur. Radiol. 32, 6435–6443. doi: 10.1007/s00330-022-08611-y 35320409

[ref43] Zhao Y. Chen J. Jiang R. Xu X. Lin L. Xue Y.. (2022). MRI features of responsible contacts in vascular compressive trigeminal neuralgia and prediction modeling. Acta Radiol. 63, 100–109. doi: 10.1177/0284185120983971, PMID: 33412924

[ref44] Zhong H. Zhang W. Sun S. Bie Y. (2022). MRI findings in trigeminal neuralgia without neurovascular compression: implications of petrous ridge and trigeminal nerve angles. Korean J. Radiol. 23, 821–827. doi: 10.3348/kjr.2021.0771 10.3348/kjr.2021.0771 PMC9340232 35695314

